# Treatment of Androgenetic Alopecia Using PRP to Target Dysregulated Mechanisms and Pathways

**DOI:** 10.3389/fmed.2022.843127

**Published:** 2022-03-16

**Authors:** Rama Abdin, Yusheng Zhang, Joaquin J. Jimenez

**Affiliations:** ^1^Charles E. Schmidt College of Medicine, Florida Atlantic University, Boca Raton, FL, United States; ^2^Dr. Phillip Frost Department of Dermatology and Cutaneous Surgery, University of Miami Miller School of Medicine, Miami, FL, United States

**Keywords:** male pattern hair loss, autologous therapy, androgenetic alopecia, hair follicle biology, hair growth, platelet rich plasma, hair loss treatment

## Abstract

Androgenetic alopecia (“AGA”) is the most prevalent type of progressive hair loss, causing tremendous psychological and social stress in patients. However, AGA treatment remains limited in scope. The pathogenesis of androgenetic alopecia is not completely understood but is known to involve a hair follicle miniaturization process in which terminal hair is transformed into thinner, softer vellus-like hair. This process is related to the dysregulation of the Wnt/β-catenin signaling pathway, which causes premature termination of the anagen growth phase in hair follicles. Historically used for wound healing, platelet rich plasma (“PRP”) has recently been at the forefront of potential AGA treatment. PRP is an autologous preparation of plasma that contains a high number of platelets and their associated growth factors such as EGF, IGF-1, and VEGF. These factors are known to individually play important roles in regulating hair follicle growth. However, the clinical effectiveness of PRP is often difficult to characterize and summarize as there are wide variabilities in the PRP preparation and administration protocols with no consensus on which protocol provides the best results. This study follows the previous review from our group in 2018 by Cervantes et al. to analyze and discuss recent clinical trials using PRP for the treatment of AGA. In contrast to our previous publication, we include recent clinical trials that assessed PRP in combination or in direct comparison with standard of care procedures for AGA such as topical minoxidil and/or oral finasteride. Overall, this study aims to provide an in-depth analysis of PRP in the treatment of AGA based on the evaluation of 17 recent clinical trials published between 2018 and October 2021. By closely examining the methodologies of each clinical trial included in our study, we additionally aim to provide an overall consensus on how PRP can be best utilized for the treatment of AGA.

## Introduction

**Figure 1:**
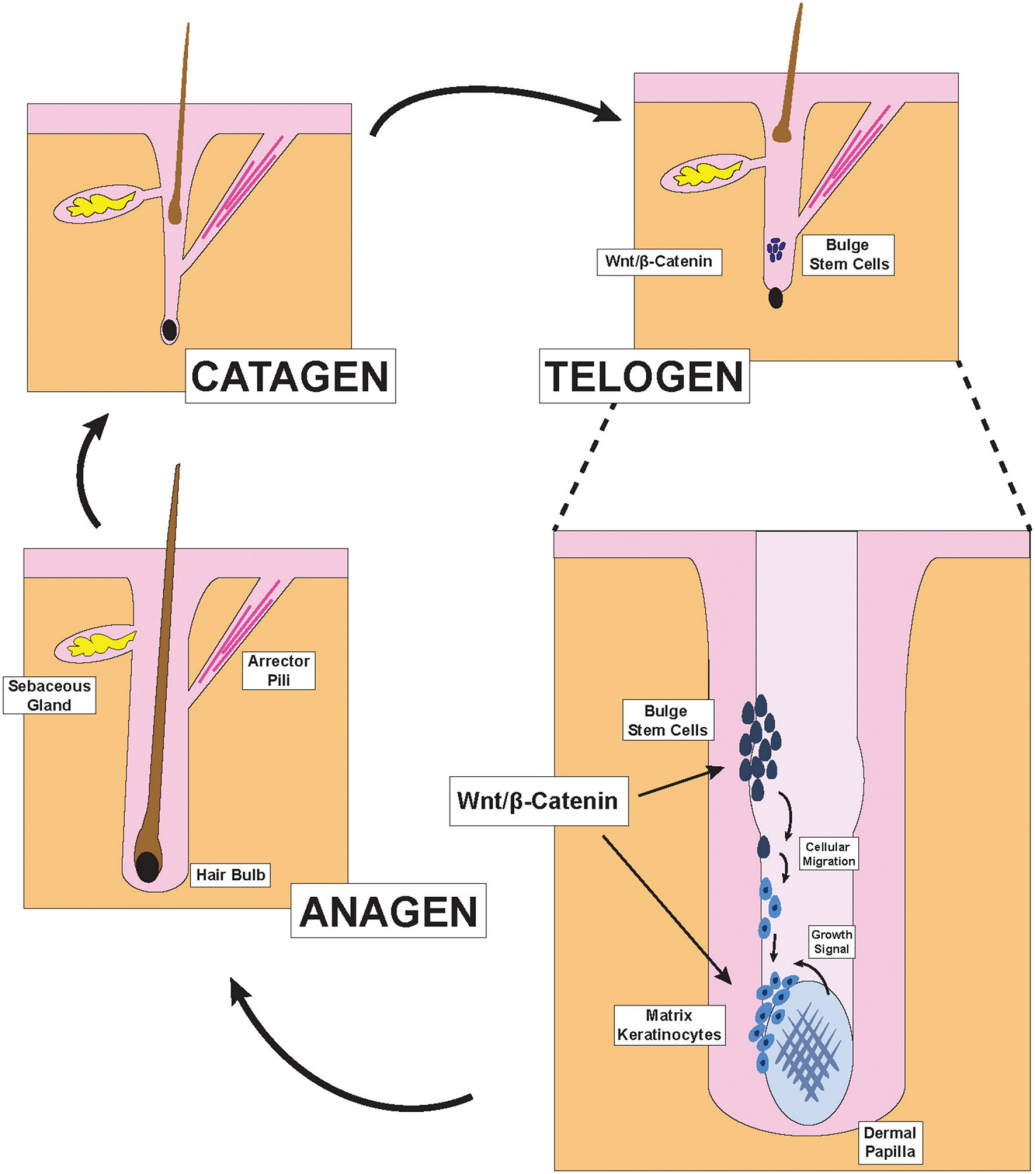
Hair Follicle structures and phases of the hair cycle. Illustration depicting pertinent structures of the hair follicle as well as the effect of Wnt/β-catenin signaling on hair formation. During the late telogen/early anagen phase, molecular signals from the dermal papilla promote the migration and differentiation of bulge stem cells to form new matrix keratinocytes of the anagen bulb.

**Figure 2:**
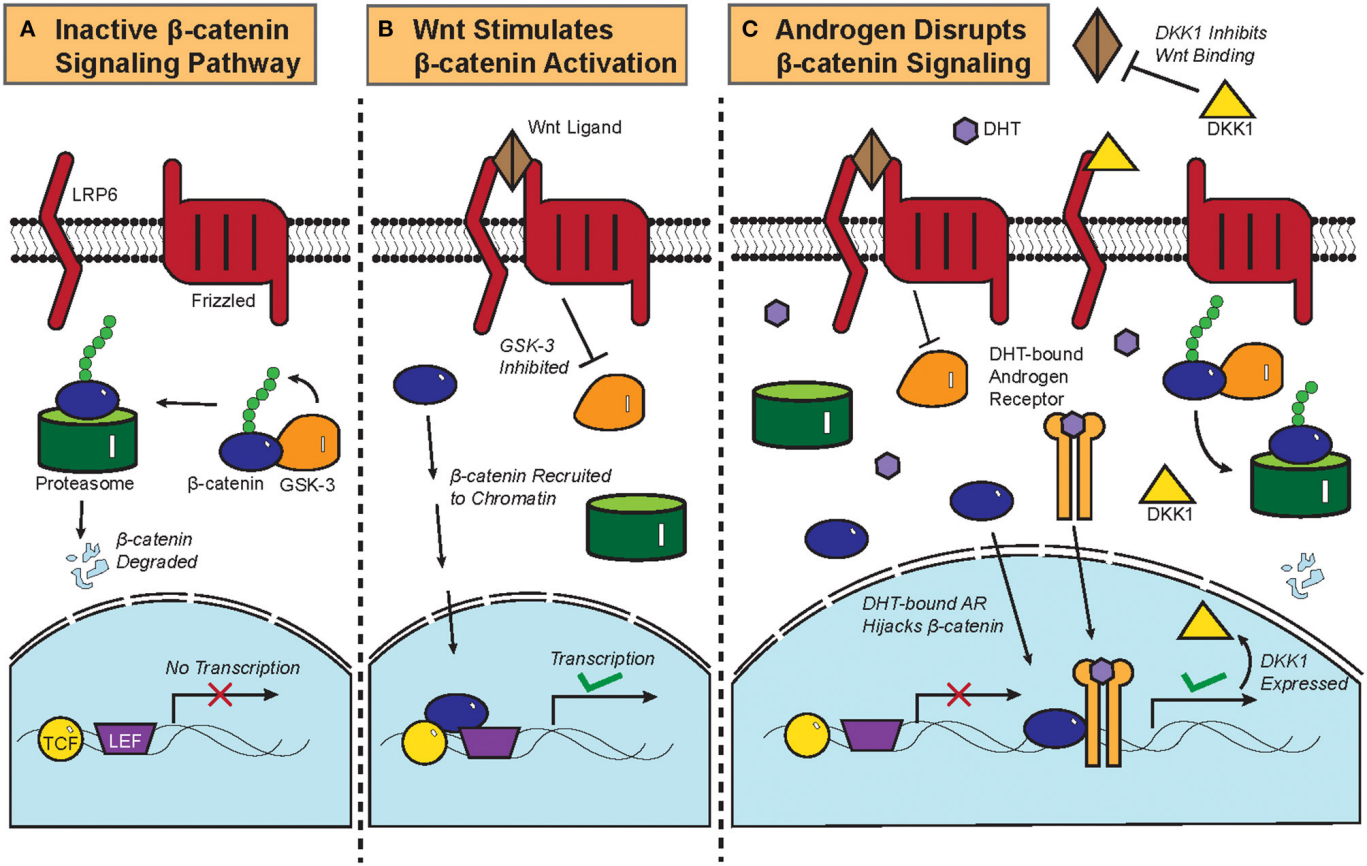
Biochemistry of the Wnt/β-catenin signaling pathway and its crosstalk with androgen. **(A)** Constitutive phosphorylation of β-catenin by glycogen synthase kinase 3B (GSK-3B) and subsequent proteasomal degradation of β-catenin in the absence of Wnt ligand. **(B)** Binding of Wnt ligand to Frizzled and Low-Density Lipoprotein-Related Protein (LRP) and the resultant inactivation of GSK-3B and its phosphorylation of β-catenin, which is then translocated to the nucleus to initiate the transcription of target genes. **(C)** DHT-activated AR can recruit β-catenin as a coactivator to stimulate the expression of DKK1, a competitive antagonist of the Wnt/β-catenin signaling pathway. Crosstalk between DHT-activated AR and β-catenin thus leads to downregulation of β-catenin target genes.

Hair follicles cycle through three primary phases of catagen, telogen, and anagen: former two phases encompass hair follicle regression and shedding while the latter represents the formation and growth of new hair (Figure 1) (1). Hair growth is a highly regulated process that is directly dependent on the β-catenin signaling pathway, which is activated by Wnt ligands (2–4). Although there are 19 distinct Wnt genes within the human genome, some Wnt gene have been characterized to play different roles in hair follicle biology (5). The β-catenin signaling pathway exhibits crosstalk with receptors from other signaling pathways such as the estrogen receptor alpha (ERα), Retinoic Acid Receptor (RAR), and the androgen receptor (AR). Specifically, the crosstalk between the β-catenin pathway and the androgen signaling pathway represents a significant mechanism through which androgens such as DHT (dihydrotestosterone) can induce AGA (androgenetic alopecia), also known as male pattern baldness (Figure 2) (6).

**Figure 3:**
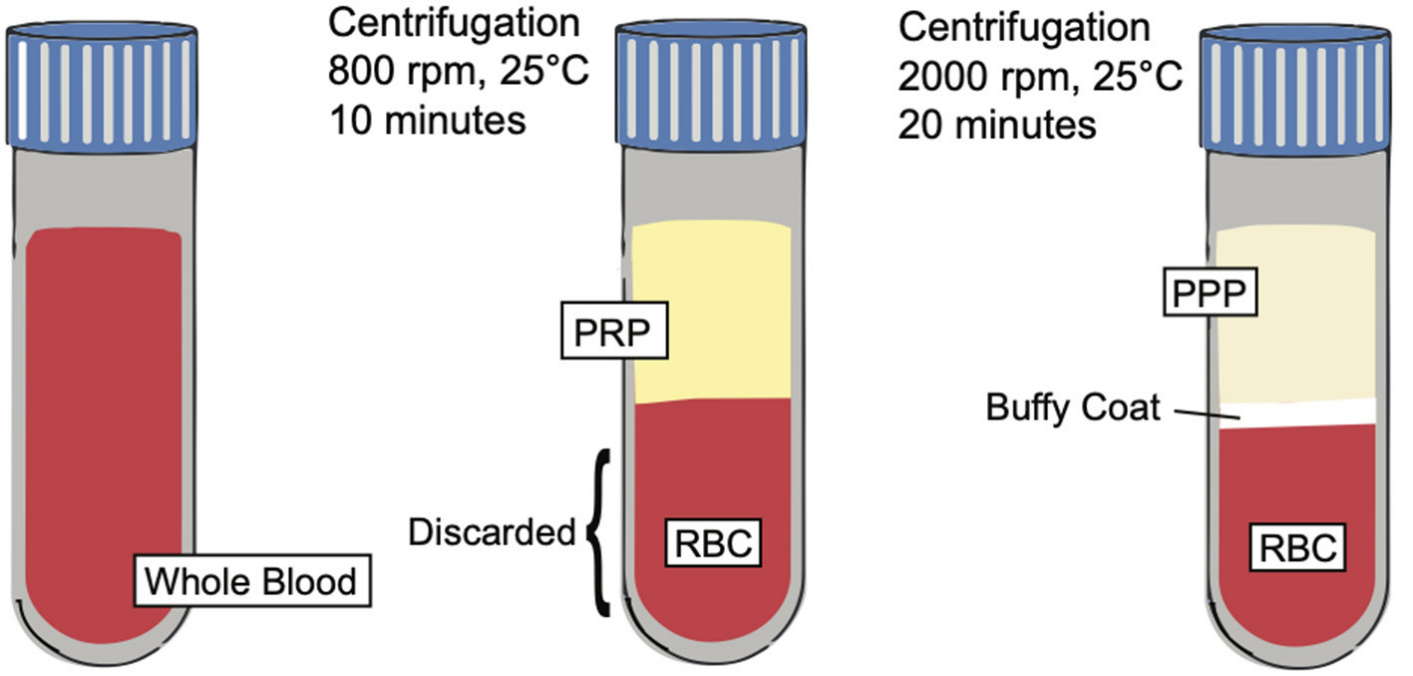
PRP preparation. Illustration depicting the preparation of PRP and showing the separation of key blood components such as platelet poor plasma (PPP), buffy coat, and red blood cells (RBCs) based on centrifugation techniques.

**Figure 4:**
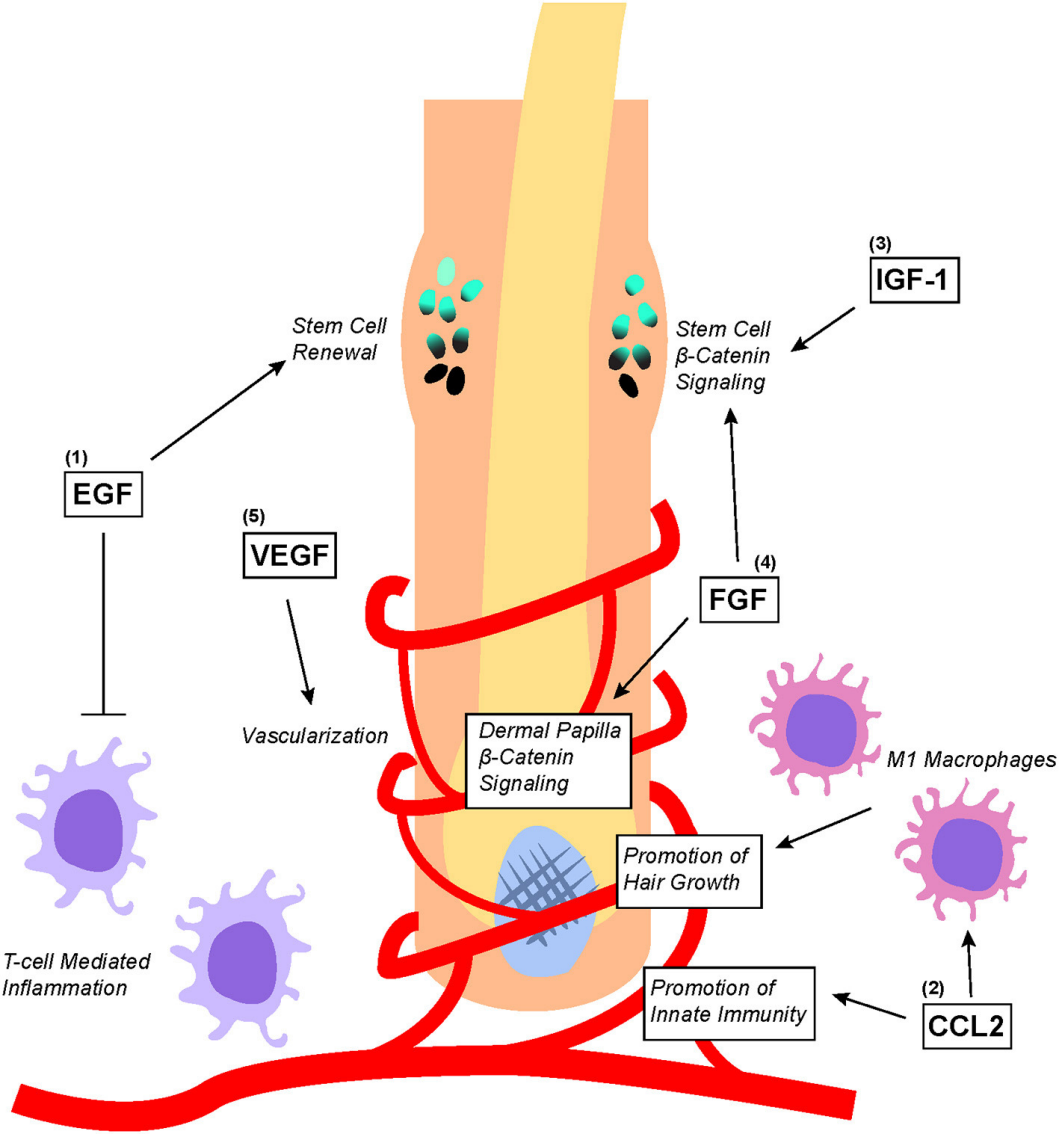
Overview of PRP-associated growth factors and chemokines as well as their effects on hair follicle biology. (1) EGF promotes bulge stem cell renewal by retarding their differentiation; additionally, EGF protects the hair follicle from excessive T-cell mediated inflammation. (2) CCL2 promotes the establishment of hair follicle-specific innate immunity as well as stimulates the recruitment and differentiation of M1 macrophages that promote hair growth. (3) IGF-1 and (4) FGF both directly upregulates Wnt/β-catenin signaling in the bulge stem cells to promote migration differentiation. Additionally, FGF stimulates Wnt/β-catenin signaling in dermal papilla to promote hair growth. (5) VEGF stimulates angiogenesis within the hair follicle microenvironment to ensure adequate nutritional delivery.

Although the majority of people experience some form of hair loss in their life time, AGA represents the most prevalent type of progressive hair loss, causing tremendous psychological and social stress in patients (7, 8). Given that the pathogenesis of AGA is driven by potent androgens such as DHT, current standard of care treatments for AGA include finasteride, which inhibit the conversion of testosterone to DHT (9). Another standard of care treatment for AGA is minoxidil, which is hypothesized to promote the delivery of nutrients and oxygen to hair follicles, thereby shortening the telogen phase (10). However, these treatments remain limited in scope, and novel treatments that are more effective and act more quickly are needed for the treatment of AGA. Although initially studied for its potential in promoting wound healing, PRP (platelet rich plasma) has been extensively studied in many recent clinical trials for its potential to promote hair growth and to reverse the signs of AGA (11). PRP is prepared from whole blood by extracting and condensing the fraction of plasma that is rich in platelets (Figure 3). By doing so, PRP thereby contains a higher concentration of platelet-associated growth factors such as EGF, IGF-1, and VEGF, each has been characterized to play an important role in promoting and maintaining hair growth (Figure 4) (12).

In this review, we begin by introducing the molecular mechanisms behind the pathogenesis of androgen-driven hair loss. Special emphasis is placed on the discussing the Wnt/β-catenin signaling pathway, the biology of androgen signaling, and how the two pathways converge. We then provide a brief introduction to hematology, leading into a deeper discussion on how PRP is extracted and produced as well as the clinical implications that come with different methodologies of PRP production. We also briefly discuss how the growth factors found within PRP impact hair follicle biology. Most importantly, we provide an in-depth analysis of all clinical trials published between 2018 and October 2021 that studied PRP as a treatment for AGA. Lastly, we provide a general consensus on how PRP can be best used for treating AGA and provide our observations regarding the clinical contexts in which PRP may be most effective.

## Hair Follicle Biology and Hair Growth Physiology

### Hair Follicle Structure, Components, and Growth Cycle

Hair is one of the primary characteristics of mammals and exerts many important physiological functions. Terminal hair, which is found on the scalp, is the subject of extensive research in dermatology because its abundance and health directly impacts the psychological well-being of many in our society (13). To generate hair strands, each hair follicle cycles through three primary phases: catagen, telogen, and anagen (Figure 1) (1). During the catagen phase, the hair follicle regresses via apoptosis and sheds the old hair. After lying dormant during the telogen phase, the hair follicle re-enters the anagen growth phase to re-initiate the formation of new hair. This process is carefully coordinated by the interactions between the ectoderm- and mesoderm-derived cells within each hair follicle and have previously been reviewed (2, 3).

Two distinct parts exist within each hair follicle: the upper portion that does not regress during the hair cycle, and the lower portion which undergoes regression and remodeling during each cycle. The anatomy of the upper portion includes the infundibulum, the opening of the hair canal to the skin, at the top; the sebaceous gland just below the infundibulum; the isthmus, where the arrector pili muscle inserts into the hair outer root sheath; and the bulge region, which contains hair follicle stem cells and is just above the junction between the upper and lower portions of the hair follicle (2). The lower cycling portion includes two key components: the anagen bulb and the dermal papilla (2). During each hair cycle, stem cells from the bulge region migrate toward the anagen bulb at the deepest level of the lower portion and give rise to activated matrix keratinocytes that colonize the matrix area, which later forms the hair shaft and parts of the root sheaths of hair (Figure 1) (2). In contrast to these cells of the ectodermal lineage, the dermal papilla is a cluster of mesodermal fibroblasts that exist below the matrix keratinocytes and form the core of the anagen bulb. Migration of the aforementioned epithelial stem cells that give rise to the activated matrix keratinocytes depend on molecular signals from the dermal papilla (3). Indeed, the size and shape of the hair strand depend on the number of dermal papilla cells within the anagen bulb. Insufficient accumulation of dermal papilla cells within the anagen bulb would fail to stimulate new hair generation regardless of keratinocyte numbers (14). The Wnt/β-catenin pathway is the primary signaling pathway through which dermal papilla dictates hair bulb size and hair shaft diameter (Figure 1) (15).

### Regulation of the Hair Cycle Depends on Wnt/β-Catenin Signaling

The biochemistry of the canonical Wnt/β-catenin signaling pathway has been previously reviewed (4). Briefly, β-catenin is constitutively phosphorylated by glycogen synthase kinase 3B (GSK-3B) and targeted for proteasomal degradation in the absence of the Wnt ligand (Figure 2A). Once the Wnt ligand binds Frizzled, a G-protein Coupled Receptor, and the Low-Density Lipoprotein-Related Protein (LRP); GSK-3B becomes inactivated and ceases to phosphorylate β-catenin. Upon translocation into the nucleus, β-catenin is recruited to chromatin with the transcription factors T-cell Factor/Lymphoid Enhancer Factor (TCF/LEF) and initiates transcriptional activation of genes (Figure 2B).

The role of β-catenin in regulating hair follicle formation was uncovered in 1998 when the ectopic expression of a degradation-resistant truncated β-catenin in mice keratinocytes promoted *de novo* hair follicle morphogenesis (16). This was followed soon after by another study which found that β-catenin signaling in chicken skin regulates the development of feather buds (17). Skin-specific ablation of β-catenin in mice abrogates the formation and development of hair, as keratinocytes that originate from the stem cell population of the hair follicle bulge lose the ability to form hair (18). Furthermore, transient activation of the β-catenin pathway in keratinocytes can induce progression from the telogen to the anagen phase, leading to active hair growth (19). Wnt/β-catenin signaling is therefore indispensable for the formation of hair follicles as well as the promotion of hair growth.

Later studies sought to elucidate the cell type-specific roles of Wnt/β-catenin signaling within the hair follicle. It was discovered that β-catenin deletion in dermal papilla cells within fully formed hair follicles reduced the proliferation of stem cells that give rise to the matrix keratinocytes within the anagen bulb. This led to thinner and shorter hair as well as premature anagen termination/catagen induction (20). Additionally, the absence of β-catenin signaling in dermal papilla cells abrogated the regeneration of hair follicles (20). Unsurprisingly, the continuous ectopic expression of β-catenin in the dermis produced larger hair follicles and accelerated the differentiation of hair follicle components (21). Additionally, it was found that epidermal Wnt ligands were required for β-catenin signaling in dermal papilla cells (21). The result was corroborated by another study showing that the ablation of Wnt ligand secretion specifically within the epidermal compartment of the hair follicle during telogen inhibited the anagen induction and resulted in hair cycle arrest (22). This is attributed to the downregulation of β-catenin signaling in both dermal papilla cells as well as in the matrix keratinocytes (22), thereby showing that epidermal Wnt ligands are required for activating β-catenin in both components of the cycling hair follicle.

There are 19 different Wnt genes within the human genome, each encoding a unique protein (5). Different Wnt ligands have different effects on hair growth. Injection of Wnt1a-enriched media into depilated mouse skin promoted the progression of hair follicles from telogen to anagen by upregulating genes essential for hair growth (23). Wnt3a treatment can maintain anagen phase growth-inducing activities of dermal papilla cells (24). Furthermore, implantation of Wnt3a-activated dermal papilla cells onto mice skin induced a more robust hair growth than control cells without prior Wnt3a activation (25). Wnt10b overexpression within the hair follicle increased the size of the hair follicles but also promoted the telogen to anagen progression of hair follicles *in vivo* (26). Indeed, treatment of individual isolated hair follicles with Wnt10b promoted an even faster rate of hair elongation than treatment with Wnt3a (27). On the other hand, Wnt5a was shown to prolong the telogen phase of hair follicles and attenuate hair growth (28).

## The Role of Androgens in Androgenetic Alopecia

### Androgens, Androgen Receptor, and Androgenetic Alopecia

Weak androgens, such as dehydroepiandrosterone (DHEA), are converted to testosterone at peripheral tissues (29). Testosterone is then further converted to dihydrotestosterone (DHT), which is a more potent form of androgen, via 5-alpha reductase (30, 31). In the hair follicle, DHEA is converted to potent forms of androgen specifically in sebocytes, fibroblasts, and dermal papilla cells (32, 33). Additionally, 5-alpha reductase expression is higher in the dermal papilla cells than in epithelial cells (34), suggesting that dermal papilla cells are a major source of DHT in hair follicles. Interestingly, the androgen receptor (AR) is also expressed in dermal papilla cells but is not detected in keratinocytes (35, 36). Aromatase, which breaks down DHT, is highly expressed in the outer root sheath of hair follicles during the anagen phase and in sebaceous glands (37).

As the name implies, androgens are the primary mediator of androgenetic alopecia (29). Androgens stimulate premature termination of the anagen growth phase in hair follicles and progressively reduces the duration of the anagen phase (38, 39). Grossly, androgens promote the miniaturization of hair follicles, transforming terminal hair into thinner and softer vellus-like hair (40). Although the level of 5-alpha reductase expression was reported to be similar between the dermal papilla cells isolated from balding and non-balding spots on the same patient (34), dermal papilla cells from balding follicles express higher levels of AR than those from non-balding follicles (41). Further evidence implicating androgen as a primary mediator of alopecia lies within the difference in hair loss presentation between men and women (42). Not only do female frontal hair follicles express AR at a level 40% lower than do male frontal hair follicles, the aromatase expression in female frontal hair follicles is also six times greater than in male frontal hair follicles (42). Patients with deficient 5-alpha reductase do not exhibit male-patterned baldness (43, 44). Most importantly, the 5-alpha reductase inhibitor finasteride demonstrates significant clinical efficacy in reducing or even reversing androgenetic alopecia (45).

### Androgens Interrupt β-Catenin Signaling to Cause Androgenetic Alopecia

Functional interactions between AR and the β-catenin pathway were previously characterized in prostate cancer. AR physically interacts with β-catenin and uses β-catenin as a co-factor to amplify the transcription of AR target genes in prostate cancer, thereby competitively inhibiting TCF/LEF-mediated transcription of β-catenin target genes (Figure 2C) (6, 46). Colon cancer cells that ectopically expressed AR also repressed β-catenin gene expression upon treatment with DHT (47), showing that the AR-β-catenin functional interaction can be found in other cellular contexts (48). In fact, β-catenin has been shown to promote and regulate the ligand-dependent transcriptional activities of other nuclear receptors such as the Retinoic Acid Receptor (RAR) and the Estrogen Receptor Alpha (ERα) (49, 50).

Dickkopf-related protein 1 (DKK1) is a physiological inhibitor of the Wnt/β-catenin pathway that is expressed more highly in balding than in non-balding portions of the scalp (51). DKK1 expression correlates with a significant reduction in anagen phase hair follicles within the scalps of patients with androgenetic alopecia (52). DHT treatment increased the expression of DKK1 in dermal papilla cells, which induces apoptosis in follicular keratinocytes (Figure 2C) (51). In an experiment where patient-derived dermal papilla cells were co-cultured with keratinocytes, treatment of the dermal papilla cells with DHT inhibited Wnt3a-induced proliferation of keratinocytes (53). Even the supernatant from DHT-treated dermal papilla cells was able to inhibit the differentiation of hair follicle stem cells, thereby showing that DHT stimulates dermal papilla cells to inhibit hair growth and follicular differentiation (54). Given that β-catenin physically interacts with AR in dermal papilla cells and that testosterone treatment of hair bulbs inhibited β-catenin target gene expression (53, 55), it can be assumed that DHT drives androgenetic alopecia at least partially by interrupting the hair follicle β-catenin signaling pathway. This is corroborated by data from recent microarray gene expression data showing that Wnt/β-catenin signaling pathway genes were noticeably downregulated in the bald frontal scalps compared to the haired occipital scalps of five men with androgenetic alopecia (56).

## Hematologic Foundations of PRP

### Introduction to Platelet Biology

Platelets, the key components of platelet rich plasma, are anuclear discoid cell fragments derived from the cytoplasm of megakaryocytes. They are about two micrometers in size and have a circulating lifespan of 7–10 days at concentrations of 150 to 400 x 10^3^ /micrometer (57). Their primary function in hemostasis and thrombosis is well-documented and understood, including the processes of platelet adhesion, activation, aggregation, and secretion. Normally, platelets circulate through the vasculature, surveying for any signs of endothelial damage, and resisting adherence to the endothelial lining through the anti-adhesive properties of the intact endothelium (58). However, upon vascular injury, platelets rapidly adhere to exposed subendothelial extracellular matrix containing various platelet receptor ligands. These ligands include collagen, von Willebrand factor (vWF), laminin, fibronectin, and thrombospondin. vWF is a large glycoprotein present within the plasma, the Weibel-Palade bodies of endothelial cells, the α-granules of platelets, and the subendothelial matrix. Once activated by endothelial injury, vWF in the endothelium exposes its binding site for GPIbα, a receptor present on circulating platelets. The fleeting associations between vWF within the vasculature and GPIbα on platelets allows for platelets to “roll” onto the area of vascular injury in close association with the exposed subendothelial matrix. During this process, other platelet receptors become activated which propagates further platelet activation and ultimately results in the platelet firmly adhering to the site of injury via multiple interactions, including interactions with collagen (59, 60). Specifically, interactions with collagen produce the most robust *in vivo* platelet activation in comparison to interactions with other factors. Once the platelet is activated, integrin αIIbβ3 receptors on the surface of platelets are upregulated and bind adhesive glycoprotein ligands, thereby recruiting additional platelets. Finally, the binding of fibrinogen to αIIbβ3 results in platelet aggregation through the crosslinking of integrin receptors on two different platelets by fibrinogen (59).

Secretion involves the release of platelet granule contents from activated platelets. The three major secretory organelles within platelets are dense granules, lysosomes, and α-granules. The dense granules are the smallest of the platelet granules and mostly contain small, non-protein molecules called adenine nucleotides in the forms of ATP and ADP. They also serve as the storage granules for serotonin, pyrophosphate, and calcium. ADP is a platelet agonist that acts as a recruitment signal for other platelets to travel to the site of vascular injury. Serotonin in platelets has a similar agonist function, but to a much lesser degree than ADP (61–63). Lysosomes are the second type of platelet granule. Lysosomes are intermediately sized granules that contain a range of digestive enzymes which become active under acidic conditions. These include glycosidases, proteases, and cationic proteins with bactericidal activity (62). Lastly, α-granules are the largest and most prevalent secretory granules found in platelets. They contain glycoproteins, growth factors, chemokines, immune mediators, and clotting factors including fibrinogen, vWF, platelet factor IV, platelet basic protein, GPIb, GPIIbIIIa, thrombospondin, multimerin, P-selectin, Glut-3, platelet derived growth factor (PDGF), vascular endothelial growth factor (VEGF), transforming growth factor β (TGF-β), insulin like growth factor (IGF), endothelial cell growth factor (ECGF), and epidermal growth factor (EGF) (62, 64). The compounds found in α-granules are involved in various biologic functions including clotting, cell growth, cell adhesion, angiogenesis, cell differentiation, and immune defense. Most importantly, they serve as the basis for the use of platelet rich plasma in the treatment of androgenetic alopecia.

### Production and Use of PRP in Clinical Settings

Platelet rich plasma (PRP) is defined as an autologous preparation of plasma that contains platelet concentrations above the baseline found in whole blood. The working definition of the therapeutic concentration of PRP is around 1,000,000 platelets/microliter in a 5-ml volume of plasma. Furthermore, PRP contains a 3-to-5-fold increase in the concentration of growth factors when compared to whole blood (12, 65). It is important to note, however, that a wide variety of variables, including the preparation, storage, and activation of PRP affect its biological activity and results, which may explain discrepant results from previous studies (66). PRP is made from a double centrifugation process of anticoagulated whole blood. The first centrifugation produces 3 layers; the bottom layer is composed of mostly red blood cells, while the two top layers (including the upper layer and thin, middle “buffy coat” layer) contain mostly white blood cells and platelets. The upper layer and superficial buffy coat are then extracted and centrifuged once more. This produces 2 layers: an upper layer, known as platelet poor plasma, and a bottom pellet layer containing the platelet rich plasma (67). However, there are variations to this technique (Figure 3), including a single centrifugation process, which may affect the therapeutic effects of PRP.

One of the main attractions associated with the use of PRP in clinical settings is its safety; as a product of autologous blood, it eliminates the risk of transmissible diseases and adverse reactions. This factor makes PRP applicable to a wide range of patients; however its cost remains a limiting factor in its widespread use. Moreover, it is hypothesized that PRP offers an advantage over treatment with exogenous cytokines; PRP releases growth factors from platelets in their native form and in an appropriate biological ratio. This is not the case with high dose, single-factor therapy of recombinant growth factor treatment (68).

PRP has been primarily used in the fields of hematology, maxillofacial surgery, periodontics (69), orthopedics, and sports medicine (70). More recently, there has been interest in the role of PRP in dermatology for wound healing, skin rejuvenation, fat graft survival, and androgenetic alopecia, which is the focus of this paper (71, 72). The mechanism of action of PRP in all of these fields is based on the processes of platelet activation and secretion. Most PRP protocols call for *in-vitro* activation of the PRP preparation before injection, usually through the addition of thrombin, calcium chloride, a combination of the two, or type I collagen. However, some physicians advocate for the injection of inactivated PRP to allow for spontaneous, native collagen-induced *in situ* platelet activation. The method of activation may also influence the rate at which the platelets in PRP release their growth-factors, ranging from immediate release to more extended release. Once activated, platelets usually begin secreting their growth factors immediately, a process known as degranulation, releasing almost 100% of their growth factors within an hour (65). Platelets may continue to release some growth factors throughout the rest of their lifespan, typically 7–10 days. This begets questions surrounding the optimal timeline of PRP injection, and may be a procedural variable along which future PRP injection protocols can be standardized. As mentioned previously, there is a wide variability in the PRP preparation and administration protocols with no consensus on which protocol provides the best results (68, 73).

## PRP Growth Factors and Chemokines Implicated in Hair Follicle Biology

Some of the growth factors and chemokines within PRP such as EGF, VEGF, CCL2, IGF, and FGF have been found to play important roles in regulating hair follicle growth (Figure 4) (12). Of these growth factors, EGF is perhaps one of the most studied. Embryonic mice lacking EGFR expression display defective hair follicle formation (74–76). However, other studies found that elevated EGFR signaling in developing mouse skin inhibited the induction of hair follicles by downregulating genes important for hair follicle lineage commitment (77). Although EGFR inhibition promoted normal hair follicle formation, hair cycle induction in fully formed hair follicles was significantly attenuated (78). To make sense of these seemingly contradictory results, it has been proposed that EGFR functions to delay differentiation and lineage commitment in hair follicles in order to maintain keratinocytes in a proliferative state (2, 3). Indeed, recent findings suggest that EGFR signaling restrains Wnt/β-catenin signaling within developing hair follicles to carefully coordinate proper hair follicle formation (79).

Interestingly, EGFR-null hair follicles transplanted onto nude mice that do not normally form a coat of hair are consumed by an inflammatory reaction within 10 weeks of transplantation (80). Closer examination of the underlying molecular process found that EGFR inhibition significantly elevated the expression of lymphocyte-recruiting chemokines that induced skin inflammation and lead to hair follicle degeneration (81). Additionally, EGF produced by T-reg cells counteracted androgen-suppressed hair growth by modulating the expression of genes such as β-catenin that are implicated in hair follicle growth (82). Therefore, in addition to coordinating proper hair follicle formation, EGFR signaling also serves to protect hair follicles from immune-mediated destruction.

Nevertheless, many studies have demonstrated the important benefits of proper immune system involvement in coordinating hair regeneration. CCL2, a chemokine found in PRP, is produced by keratinocytes in the isthmus of hair follicles to regulate the renewal of Langerhans cells, which are epidermal dendritic cells important for innate immunity within the hair follicle microenvironment (83). Stressed hair follicle keratinocytes also produce CCL2 to recruit M1 Macrophage around hair follicles to promote hair regeneration by stimulating telogen to anagen phase transition (84). Macrophages recruited by CCL2 are found to secrete factors such as VEGF, IGF1, and FGF2/FGF10 (all of which are contained within PRP) to mediate hair follicle regeneration and the growth of hair (85).

Indeed, other growth factors such as IGF-1, GFG, and VEGF have also been shown to play significant roles in hair biology. In an experiment co-culturing outer root sheath cells and beard dermal papilla cells, IGF-1 secreted from beard dermal papilla cells was found to promote follicular epithelial cell growth (86). Additionally, IGF and EGF co-treatment stimulated the transition of hair follicles from the telogen to anagen phase, thereby enhancing the proliferation of outer root sheath and dermal papilla (87). FGF can also promote the induction of the anagen phase in hair follicles by stimulating the β-catenin signaling pathway (88). This correlates with earlier studies showing that the downregulation of FGF receptors significantly reduced mouse hair follicle density (89). Lastly, overexpression of VEGF by outer root sheath keratinocytes induced increased vascularization around hair follicles, which accelerated hair regrowth after depilation and increased the size of hair follicles as well as the thickness of the hair shaft (90). This was corroborated by another alopecia study showing that VEGF topical gel application for 15 days increased hair density as well as the diameter of hair shafts (91).

However, application of 100% VEGF led to significant hematological alterations as well as elevated liver enzymes (91). This shows that topical application of concentrated exogenous growth factors can produce unwanted deleterious effects, thereby highlighting the benefit of using an endogenously obtained source of growth factors such as PRP. Additionally, based on studies discussed above, hair follicle growth is dependent on the careful coordination of many growth signaling pathways. The use of PRP to treat androgenetic alopecia is hypothesized to be superior to using an artificial mixture of exogenous chemokines and growth factors because the combination of growth factors and chemokines within PRP is balanced at physiological ratio, thereby echoing our above-mentioned point.

## Overview of Recent Clinical Trials Using PRP for the Treatment of AGA

### Methodology

This study follows the previous review from our group in 2018 by Cervantes et al. to analyze and discuss recent clinical trials using PRP for the treatment of AGA. A search on PubMed/MEDLINE in October 2021 using the keywords “Platelet-rich plasma AND alopecia AND hair loss” identified 23 clinical trials performed between the years 2018 and 2021. We also included trials that assessed the efficacy of PRP in comparison or in combination with standard of care procedures for AGA such as topical minoxidil and oral finasteride. We excluded four trials that evaluated alopecia areata as well as one that examined female pattern hair loss. Additionally, we excluded the 2020 study by Butt et al. and the 2020 study by Kapoor et al. Specifically, the conclusions drawn by the authors were not consistent with the data provided within the studies. Overall, seventeen clinical trials in phases II to IV were reviewed by three authors and were summarized in Supplementary Tables 1–3. Clinical trial numbers are also provided for the studies that were performed in the U.S. and/or registered on ClinicalTrials.gov. Of the seventeen studies, excluding the two by Kapoor et al. and Butt et al. fifteen were evaluated and discussed within this narrative review (92–108).

### PRP Is an Effective Treatment for AGA

Optimizing the use of PRP for treating AGA requires further examination of the effects of PRP administration protocols on clinical outcomes. Additionally, the proposed mechanisms of action of PRP and the clinical relevance of PRP activation need to be further investigated. Hausauer et al. sought to evaluate the effects of PRP treatment protocols in the treatment of AGA in a randomized, blinded clinical trial (NCT02999737) that involved 30 men with stage II-V AGA (Norwood-Hamilton Scale) and 10 women with stage I-III AGA (Ludwig Scale) (92). The patients were split into 2 PRP protocol groups: group 1 (20 patients) received 3 monthly PRP treatments with a booster 3 months later while group 2 (20 patients) received a total of 2 PRP treatments spaced 3 months apart. Both groups received subdermal PRP aliquot injections. One patient in group 2 was lost to follow up therefore results were reported on 39 patients. Hair count and caliber, in addition to overall severity ratings, were assessed using global photography and dermoscopy at 3 and 6 months after the first PRP injection. The results of this study showed that at 3 months, only group 1 showed a statistically significant increase in hair count while group 2 did not yield a significant increase in hair count until 6 months after the first injection when compared to baseline. Furthermore, group 1 produced significantly better results than group 2 in terms of absolute and percent change between the groups. Mean hair caliber was increased similarly in both groups at 3 and 6 months. The results of this study show that more frequent PRP treatment leads to improved temporal and quantitative clinical effects, with 3 monthly PRP treatments plus a booster yielding faster and better results than 2 individual PRP treatments at 3-month intervals.

Rodrigues et al. investigated the proposed mechanism of action of PRP by looking into the relationship between PRP platelet concentration, the levels of growth factors, and the resultant hair growth (93). In this double-blind controlled study, 26 patients with vertex profile-based stage III AGA (Norwood-Hamilton scale) were split into 2 groups: group 1 (15 patients) received a total of 4 PRP treatments at 15-day intervals while group 2 (11 patients) received saline solution injections in a similar manner. Number of hairs, hair density, percentage of anagen and telogen hair, and percentage of vellus and terminal hair were assessed using dermoscopy. PRP preparations contained a 5-fold increase in the median number of platelets when compared to peripheral blood and were activated with autologous serum. Nonetheless, no correlation was found between platelet count and hair growth (*p* = 0.123 for hair count, *p* = 0.086 for hair density, *p* = 0.171 for percentage of anagen and telogen hairs, and *p* = 0.174 for percentage of vellus and terminal hairs). In addition, even though EGF and PDGF revealed a significant correlation with platelet number in PRP, there was no correlation found between these growth factors and hair count, hair density, percentage of anagen and telogen hairs, or percentage of vellus and terminal hairs. Similar results were obtained for VEGF. However, clinical evaluation of the PRP group showed a significant increase in hair count and density 3 months after the last injection. Although percentage of anagen hairs was significantly increased during treatment, this was not maintained by 3 months after the last injection. Similarly, the significant decrease in percent of telogen hairs after treatment was not sustained during the follow up. The results also showed that there was no significant improvement in the terminal to vellus hair ratio in either treatment group (*p* = 0.955 for PRP group and *p* = 0.206 for control group). Although the authors concluded that the use of PRP is efficacious in the treatment of AGA, the role of growth factors in the hair-promoting mechanisms of PRP requires further investigation.

Gentile et al. performed a retrospective, randomized, blinded study to evaluate the effects of PRP activation on hair growth (94). The study consisted of 63 men with grade I-V AGA (Norwood-Hamilton scale) and 27 females with grade I-III AGA (Ludwig scale) who were split into two groups: group 1 (57 patients) were treated with NA-PRP (non-activated PRP) while group 2 (33 patients) were treated with A-PRP (autologous activated PRP). Of these 90 patients, 3 were excluded and 1 was rejected with results being reported on 86 patients. Placebo was injected into appropriate control areas that did not encompass the entirety of half a scalp. Each group received 3 treatments spaced apart by 30 days on average. Trichospic evaluation showed an improvement in mean hair density of 23.3 ± 3 hairs/cm^2^ in group 1 and 13 ± 3 hairs/cm^2^ in group 2, 58 weeks after the last injection. These results were also statistically significant when compared to control. This study also investigated the use of micro-needling with A-PRP injection. The combination of micro-needling and A-PRP appears to have a favorable effect on hair growth with follow-up in progress. In addition, microscopic and immunohistochemistry evaluation of both NA-PRP and A-PRP-treated scalps showed a significantly increase in the number of follicles, Ki67+ basal keratinocytes, and follicular bulge cells when compared to baseline. Furthermore, both treatment groups were associated with increased vascularization of hair follicles and increased epidermal thickness of the scalp. This study demonstrates that both activated and non-activated PRP are effective in treating AGA. Importantly, the improved hair growth seen with non-activated PRP suggests that *in-vivo* activation of PRP activation may be more effective than *in-vitro* PRP activation.

A 2019 randomized clinical trial by Bayat et al. investigated the effectiveness of PRP in the treatment of AGA in men (95). 19 patients with grade III-V AGA (Norwood-Hamilton scale) were injected subcutaneously with PRP in three sessions at 4-week intervals. Improvement was quantified via digital photography and dermoscopy photos, through which the number of hair follicles and the average hair thickness per unit area were assessed by a dermatologist. In addition, clinical transformation was evaluated by two independent observers. The results showed a statistically significant increase in average hair thickness by week 4, which was maintained until 3 months after the last injection. Interestingly, the increase in hair thickness occurred predominantly after the first injection, and no significant increases in hair thickness occurred between week 8 and 3 months after the first injection. Furthermore, although the number of hair follicles significantly increased by weeks 4, 8, and 3 months after the last injection; no additional increases in hair follicle numbers were observed between week 8 and 3 months after treatment. Importantly, evaluation by observers revealed a statistically significant perceived clinical improvement. This small clinical trial demonstrates yet again the efficacy of PRP in the treatment of AGA yet calls for additional studies with larger sample sizes and longer follow up periods to better evaluate the long-term effects of PRP.

The effectiveness of PRP in the treatment of AGA in both men and women was assessed once more in a 2019 clinical trial by Butt et al. (96). Twenty males type III-VI AGA (Norwood-Hamilton scale) and ten females type I-III AGA (Ludwig scale) were treated with a total of 2 PRP treatments spaced 4 weeks apart. Clinical improvement was assessed with macroscopic photographs, pull test, trichoscopy, a physician global assessment score, and a patient global assessment score. The study notes that in most patients, terminal to vellus hair ratio was increased, however it also remained static in others. Furthermore, mean hair density and pull test results were significantly improved by 6 months after the 1st injection. These improvements were corroborated by both physician and patient global assessment scores- indicating satisfactory perceived improvements in hair growth with the use of PRP. The results of this study offer additional data in support of the use of PRP for treating AGA in men and women.

Dubin et al. conducted a randomized, blinded, controlled study (NCT03689452) published in 2020 to assess the effectiveness of PRP in the treatment of AGA in women (97). In this study, 30 women with grade I-III AGA (Ludwig scale) were split into 2 groups: 15 women were treated with subdermal PRP injections in three sessions with 4-week intervals, and the other 15 women received saline injections in an identical fashion. One patient was lost to follow up in each group, therefore results were reported on 14 patients each in the PRP group and the placebo group. Mean hair density and shaft caliber were assessed using global photography and dermoscopy photos. Statistically significant improvements in mean hair density and caliber were seen in the PRP group vs. placebo group at week 8 when compared to baseline, and this enhancement was maintained at 24 weeks. In addition, blinded global photographic assessment revealed a significant improvement in PRP group patients from baseline. The results of this study lend additional support to the efficacy of PRP in treating AGA.

A recent randomized, placebo-controlled, half-head, double blind study in 2021 by Qu et al. further substantiated the clinical efficacy of PRP in the treatment of AGA (98). 32 male patients with stage II-V AGA (Norwood-Hamilton scale) and 20 female AGA patients with stage I-III AGA (Ludwig scale) were subdermally injected with PRP on one half-head of the affected scalp area while the other half-head were injected with saline solution. The patients were treated in three consecutive sessions with 1-month intervals. It was found that at 3 and 6 months, mean hair count, density, and diameter all showed a statistically significant improvement when compared with baseline, and the anagen hairs ratio was noted to be increased by 6 months. When compared to the control, PRP proved to be effective by showing significant improvement in hair density starting at 3 months, and improvements in hair count, diameter, and anagen hair ratio at 6 months. In addition, macrograph assessment by five independent experts revealed a significant perceived improvement in hair growth on the PRP treatment side when compared to the control side. Lastly, an end-of-study satisfaction questionnaire yielded a mean satisfaction score of 4.23/5 across the entire study period, indicating satisfaction by the patients treated with PRP. The results of this study further support the clinical effectiveness of PRP in treating AGA and demonstrate the potential for quality-of-life improvement with PRP treatment as evidenced by high patient satisfaction scores.

### PRP Acts Synergistically With Topical Minoxidil and Oral Finasteride to Promote Hair Growth

Many important questions still remain unanswered in the field of AGA research. Key amongst those are (1) whether PRP is more effective than standard of care (Minoxidil and Finasteride) in reversing hair loss and (2) whether the effects of PRP can be compounded with that of minoxidil and finasteride to increase hair growth. In an attempt to address these questions, Alves et al. (99) in 2018 conducted a randomized controlled double-blind half-head clinical trial involving 11 male patients with grade II-V AGA (Hamilton-Norwood Scale) and 13 female patients with grade I-III AGA (Ludwig Scale). For a duration of 3 months, half the patients received 1 mg of oral finasteride daily while the other half received twice-daily topical 5% minoxidil application. Each patient's scalp was further divided into halves – one side received three doses of monthly PRP injections while the other side was injected with saline. The results showed that under concurrent minoxidil or finasteride treatment, the PRP-treated side exhibited significantly greater hair count and density when compared to the place-treated side. Interestingly, despite receiving minoxidil or finasteride treatment, the side of scalp that received placebo injections did not exhibit increased hair count or density when compared to baseline. Failure to demonstrate significant therapeutic efficacy using standard of care treatments (positive control) brings much skepticism toward the validity of the study's methodology. Nevertheless, it was shown with statistical significance that PRP administration can promote hair growth alongside concurrent topical minoxidil or oral finasteride use in patients with AGA.

The hair growth-promoting effects of PRP were again compared to that of topical minoxidil in a 2019 randomized double-blind controlled clinical trial by Singh et al. which enrolled 80 male patients with grade II-IV AGA (Hamilton-Norwood Scale) (100). Patients were divided into four groups; and for a period of 3 months, Group 1 patients received twice-daily topical applications of 5% minoxidil with monthly placebo (saline) injections; Group 2 received 5% topical minoxidil along with monthly PRP injections; Group 3 received topical saline along with monthly placebo injections; and Group 4 received topical saline with monthly PRP injections. The authors found that minoxidil treatment significant improved hair density after 3 months of treatment. However, PRP treatment significantly improved hair density after only 1 month regardless of whether patients were concurrently receiving minoxidil. Although minoxidil treatment significantly increased hair density upon the conclusion of the study, treatment with both minoxidil and PRP was significantly more effective than just minoxidil alone. Additionally, PRP treatment alone appeared to be more effective than minoxidil alone, and the difference trended toward significance (*p* = 0.076). Overall, patients in Group 2 (PRP/minoxidil) had higher hair density than patients in Group 4 (PRP) who in turn had higher hair density than patients in Group 1 (minoxidil), thereby conclusively showing that the therapeutic effects of PRP and minoxidil can be compounded.

The results of Singh et al. were corroborated by Pakhomova et al. in a randomized single-blind controlled clinical trial that involved 69 men aged 18–53 with grade I-IV AGA (Hamilton-Norwood Scale) (101). Subjects were divided into three groups; and for a period of 4 months, group 1 patients received monthly PRP injections, group 2 patients received monthly PRP injections as well as twice-daily topical 5% minoxidil treatment, and group 3 patients only received twice-daily topical minoxidil. Upon conclusion of the study, the authors found that all three treatments significantly increased hair density, with the combined topical minoxidil/PRP treatment increasing hair growth more than either treatment alone. The therapeutic advantage of the combined treatment over either monotherapy was evident in other objective measurements such as the decline in percentage of vellus hair, average hair diameter, and the decline in share of telogen hair. Although minoxidil and PRP monotherapies alone produced similar increases in hair density, PRP was significantly more effective than minoxidil in increasing average hair diameter, decreasing the percentage of vellus hair, and decreasing the percentage of hair in the telogen phase. In addition to showing again that the therapeutic effects of PRP and topical minoxidil can be combined, these results suggest that PRP is more effective than minoxidil in promoting the growth of thick, terminal hair.

Most recently, a single-blinded, randomized, controlled study by Ramadan et al. Sought to evaluate the effect of PRP application technique on hair growth in the treatment of AGA (102). Additionally, the study incorporated minoxidil and finasteride treatments. 46 male patients with stage II-IV AGA (Norwood-Hamilton scale) and 80 female patients with stage I-III AGA (Ludwig scale) received combined therapy consisting of topical 5% minoxidil (once daily in females and twice daily in males) and oral hormonal therapy (spironolactone in females and finasteride in males). 84 of the aforementioned patients were selected to receive PRP treatment and were then further split into 2 groups: group I (42 patients) received direct syringe-based intradermal PRP injection, and group II (42 patients) underwent automated PRP application with micro-needling. Patients in each group were treated with 3–6 sessions at 1-month intervals; the degree of improvement was evaluated after three sessions; and if improvement was seen, monthly treatments resumed for an additional 2–3 sessions. The remaining 42 patients who did not receive PRP were control. Progress and results were evaluated with hair pull test, macrograph, and dermoscopy. The clinical improvements were evaluated by 3 dermatologists in a blinded fashion. Overall, when compared to control, both groups I and II showed significant clinical improvements, with group II showing significantly greater improvements than group I. Group II patients also demonstrated significant improvements in hair density and diameter while group I patients did not (*p* = 0.092 for hair density, *p* = 0.129 for diameter). This difference between patients in groups I and II was statistically significant. Pull test became negative in over 95% of patients treated with PRP after 6 months. The majority of patients treated with PRP reached best improvement in 6 months while those in the control (combined therapy only) improved after 11–13 months. Patient satisfaction showed improvement after three sessions of PRP injection, but not until at least 6 months for the control group. The results of this study indicate that automated micro needling injection of PRP is more effective than intradermal injection of PRP by syringe in the treatment of AGA. Additionally, this study again shows that the addition of PRP provides more rapidly-effective therapeutic outcomes than using the standard of care therapies alone in the treatment of AGA.

A smaller study published in 2020 by Bruce et al. (NCT03488108) attempted to examine and compare the efficacies of PRP and topical minoxidil in women with AGA (103). Twenty women aged 18 or older with grade I-II AGA (Ludwig Classification) were enrolled in a randomized controlled clinical trial employing a complicated cross-over design. Patients were divided into arms A and B with 10 patients in each arm. Patients in Arm A received 3 sessions of PRP injections spaced 1 month apart for the first 12 weeks. After a washout period of 8 weeks, these patients then received once-daily topical application of 5% minoxidil per manufacturer guidelines for another 12 weeks. Arm B patients were instead treated with minoxidil in the first 12 weeks and PRP injections in the last 12 weeks. Patients were assessed at baseline, week 12, week 32, and week 48. Compared to baseline, Arm A patients treated with PRP showed a 7.7% increase (*p* = 0.002) in hair count at week 12. However, the increase in hair count was 22.7% (*p* < 0.001) for Arm B patients who were treated with topical minoxidil, significantly higher than for Arm A patients (*p* = 0.009). Additionally, Arm B patients showed significant increases in terminal hair density and cumulative hair thickness whereas Arm A patients exhibited no significant change. Following a washout period of 8 weeks and a second round of switched 12-week treatment, patients in Arm A showed a 37.4% increase (*p* = 0.004) in hair count at week 32 after switching to minoxidil whereas patients in Arm B showed a smaller hair count increase of 20% (*p* = 0.020) after switching to PRP. Patients in Arm A also showed significant increases in other objective measurements such as terminal hair density and cumulative thickness whereas patients in Arm B exhibited no change. Although this clinical trial enrolled fewer patients than the two that were previously discussed, the results seem to suggest that minoxidil may be more effective than PRP in treating signs of AGA in women. This is in stark contrast to the studies by Singh et al. and Pakhomova et al. which seem to show that PRP is more effective than minoxidil in treating AGA in men. Interestingly, at week 48 (4 months after conclusion of treatment), only Arm B patients who were last treated with PRP maintained a significant increase in hair count when compared to baseline. Patients in Arm A who last received minoxidil seemed to have lost all therapeutic gains in hair growth, therefore suggesting that the hair promoting effects of PRP may either take time to manifest in women and/or are more enduring than that of minoxidil.

### Assessment of Studies That Demonstrate PRP Is Ineffective in Treating AGA

One of the recent studies that call into question the efficacy of PRP in treating AGA is a study by Shapiro et al. from 2020 (104). This randomized, double-blind, controlled clinical trial (NCT02591355) enrolled 18 men with grade III-V AGA (Norwood-Hamilton Scale) and 17 women with grade I-II AGA (Ludwig Scale) between the ages of 18 to 58. Using a half-headed experimental design, patients were treated with three once-a-month PRP injections on one side and placebo on the other. Patients were examined at baseline and at 1, 2, and 4 months (2 months after last PRP injection). PRP-treated scalps showed a 13.2% increase in hair density at 5 months (*p* < 0.05). However, placebo-treated scalps showed similarly significant increases in hair density. Additionally, both PRP and placebo treatments seemed to increase hair diameter to a similar degree. The authors attributed the equivocal findings to a possible diffusion effect whereby PRP from the treatment side may have eluted to the placebo side to induce hair growth. However, it is also important to note that this smaller-scale study employed almost-equal numbers of subjects from both sex. This is in contrast to the previously mentioned larger-scale studies by Gentile et al. and Que et al. that enrolled more men than women. Given that PRP may be more effective for the treatment of AGA in men than in women per results of studies discussed above, employing similar numbers of subjects from both sex may have contributed to the inconclusive findings. It is also possible that this study with only 35 subjects did not have sufficient power to detect significant differences between treatment and control. Nevertheless, objective scalp assessment by independent expert reviewers found that a greater proportion of scalp areas treated with PRP showed clinical improvement than those treated with placebo.

Another study from 2020 also found that treatment with PRP had no effects on promoting hair growth in patients with AGA. Gressenberger et al. (105) enrolled 30 men aged 18–52 with grade III or higher AGA in a randomized, single-blinded, controlled clinical trial. Twenty subjects were treated with five injections of PRP spaced 4 to 6 weeks apart while ten received placebo injections. Subjects were assessed at baseline, at 1 month after final treatment, and at 6 months after final treatment. Despite the high number of PRP treatments (five here vs. the typical three in previously-discussed studies), the authors found that PRP did not increase the median hair density nor median hair diameter at any point during the trial. Independent review of visual improvement also failed to find any improvement in hair growth with PRP treatment. It is interesting to note that in contrast to all other studies discussed previously, Gressenberger et al. measured the median instead of mean hair density and hair diameter. A mean value is the average of all measured values whereas a median value denotes the exact halfway between the lowest and the highest values. It is entirely possible that the portion of scalp areas that responded to PRP treatment is similar to the portion that did not respond, thereby resulting in an unchanged median value. Additionally, the authors stated in their protocol that the amount of PRP administered differed “depending on the degree of AGA,” which is another aspect of their trial protocol that differed from the other previously discussed clinical trials.

The last recent clinical trial that failed to show significant improvements in hair growth parameters upon PRP administration is by Siah et al. (106). This randomized, single-blind, half-headed, controlled clinical trial enrolled a total of 10 patients (9 female, 1 male) aged 20–55 with clinically diagnosed AGA of undisclosed grade/severity. Every patient was administered five injections of PRP spaced 2 weeks apart on one-half of the scalp while the other half received placebo. Patients were assessed at baseline, at 1 month (after 2 injections), and at 4 months (2 months after last injection). Although PRP treatment increased average hair density by 12.7% at 4 months, the increase was not statistically significant. Unexpectedly, both PRP and placebo treatments decreased hair diameter at 4 months, although the change was also not statistically significant. As previously discussed, perhaps the use of many more female than male subjects in this clinical trial contributed to the inconclusive findings of this study. Additionally, the small number of subjects enrolled in this study may have failed to provide enough power to the study to draw statistically significant conclusions. Nevertheless, the authors found that the final platelet enrichment in PRP is significantly correlated with the platelet concentration in peripheral blood, thereby showing that future clinical trials using PRP may require stringent quality standardization.

## Discussion

The hair cycle is a carefully coordinated process that is heavily dependent on the Wnt/β-catenin signaling pathway. However, this pathway is prone to dysregulation by other physiological factors, namely androgen signaling, thereby leading to pathological hair loss known as AGA. For both men and women worldwide, androgenetic alopecia brings significant social and psychological stress. Although standard of care treatments (namely topical minoxidil and oral finasteride) can halt or even reverse the signs of AGA; novel, more effective, and faster-acting therapeutic strategies are needed. PRP has been heavily investigated in recent years as a possible treatment for AGA due to its vast yet understudied therapeutic effects. By containing a platelet concentration that is several-folds greater than that of physiological plasma, PRP thereby also contains various growth factors such as EGF, FGF, IGF, and VEGF at supraphysiologic concentration yet also at physiologic proportions. Each of these factors activate important biological mechanisms that promote hair growth by either directly affecting the Wnt/β-catenin signaling pathway or by establishing a permissive microenvironment for healthy hair follicles to take root. Recently clinical trials have mostly shown that PRP treatment is highly effective in treating AGA. Not only can the hair-promoting effects of PRP be compounded with that of standard of care treatments (minoxidil, finasteride), many clinical trials comparing PRP against standard of care treatments have shown that PRP is more effective and more rapidly-acting than topical minoxidil and oral finasteride.

Nevertheless, there are few recent clinical trials with data that continue to suggest PRP may not be effective at all as a treatment for AGA. Furthermore, a conclusive link between growth factor concentrations in PRP and the rate of hair growth has not yet been demonstrated. Future studies should continue examining the hair-promoting mechanisms of PRP while paying close attention to the growth factors contained within PRP. Additionally, the hair-promoting effects of PRP that are unrelated to growth factor concentrations should still be explored.

## Author Contributions

JJ and YZ contributed to conception, design, and review of this work. YZ, RA, and JJ created the figure design. YZ and RA reviewed the literature and wrote the manuscript. All authors contributed to the article and approved the submitted version.

## Conflict of Interest

The authors declare that the research was conducted in the absence of any commercial or financial relationships that could be construed as a potential conflict of interest.

## Publisher's Note

All claims expressed in this article are solely those of the authors and do not necessarily represent those of their affiliated organizations, or those of the publisher, the editors and the reviewers. Any product that may be evaluated in this article, or claim that may be made by its manufacturer, is not guaranteed or endorsed by the publisher.
